# Selective decontamination of the digestive tract in critical care: a teenage angst or coming of age issue?

**DOI:** 10.1186/s13054-018-2227-2

**Published:** 2018-11-14

**Authors:** Brian H. Cuthbertson

**Affiliations:** Department of Critical Care Medicine, Sunnybrook Health Sciences Centre, University of Toronto, Toronto, M4N 3M5 Canada

**Keywords:** Sepsis, Prevention, Selective decontamination of the digestive tract, Antibiotics, Evidence

## Abstract

Selective decontamination of the digestive tract (SDD) has been with us since the early days of our specialty, and in some ways it marks our progression and maturation. How we have dealt with SDD to date ranges from “thorn in our side” to “elephant in the room”. With high quality multi-national studies underway, how we deal with these results will mark our final maturation to adulthood as a specialty.

It is said that that there are several ages of man: infancy, adolescence, coming of age, adulthood and senility or as Douglas Adams stated Survival, Inquiry and Sophistication, otherwise known as the How, Why and Where phases [1]. It could be suggested that the stages of man have their analogies for a medical specialty, and if this holds true, then intensive care medicine should surely be passing from its coming of age stage into adulthood at this time in its development. Since selective decontamination of the digestive tract (SDD) has been with us through all stages to date, perhaps how we deal with this issue is a litmus test of whether we have indeed reached adulthood as a profession.

Back in the 80s, when SDD first came to eminence (or was it notoriety), we were still an infant specialty. At this time, we correctly identified that sepsis was “our disease” and we needed to be better at both preventing and treating it to save more lives. This led to the suggestion that using topical antibiotics to selectively target Gram-negative aerobic bacteria in the gut (the cause of the majority of hospital-acquired infections at that time) could prevent sepsis; from this SDD was borne [2]. Since then there has been over 37 randomised controlled trials (RCTs) and 12 meta-analyses (more meta-analyses than many topic areas have individual RCTs) [3]. In brief, these meta-analyses suggest that an SDD regimen that includes an intra-venous antibiotic saves lives and prevents ventilator associated pneumonia (VAP) in the critically ill [3]. Further, meta-analysis of the (mostly inadequate) antibiotic resistance data arising from these RCTs suggests that SDD may have no effect, or potentially reduce antibiotic usage and antibiotic resistance rates [4]. Despite this, detailed surveys and studies of barriers to implementation show that a large number of centres around the world have neither implemented SDD into their practice nor intend to do so [5]. The reasons quoted for this stance ranged from considered and reasonable (“I am concerned about antibiotic resistance”) to extra-scientific (“there is no supportive evidence” and “in order to adopt SDD in my unit someone would have to assassinate me”), with extra-scientific in this context clearly being a gentle euphemism for biased [6].

So why does SDD bring about such strong reactions amongst our profession and why has there been so little implementation of this strategy into our practices? There is no question that rising antibiotic resistance rates now threaten our ability to treat infections with antibiotics. There is also little doubt that, despite the large number of RCTs in this field, the age and spectrum of methodological quality of these RCTs makes the strength of evidence less than conclusive [3]. Further, with the implementation of various strategies to reduce infectious complications in critical care, the contemporary relevance of this evidence base may also be in doubt. If these were the reasons by which we had conscientiously and considerately declined to implement SDD into our practice, we would be largely justified; but there is more to this story. The evidence would suggest that our biases towards SDD may have clouded our judgement [6]. We could argue that we are a conservative specialty that appropriately awaits rigorous evidence before considering clinical implementation; and that would be laudable if it were true. However, as a profession we have implemented other treatments with far less supportive evidence, including the widespread implementation of steroids in septic shock after a small RCT of moderate quality [7, 8]; the promotion and implementation of chlorhexidine mouthwash for VAP in general critical care populations by various governmental and non-governmental bodies despite a weak to moderate evidence base coming mostly from trials in cardiac surgery patients [9]; the promotion and widespread implementation of tight glycaemic control despite the evidence coming from one single centre RCT of moderate quality [10]. All of these areas were succeeded by higher quality evidence that demonstrated that these treatments were either harmful or at least non-beneficial [11–13]. Not so conservative, it would seem!

So, returning to our analogy, these examples seem to show the teenage angst of our profession as we struggle to deal with developing, and at times contradictory, evidence bases. Going forward we need to deal with evolving evidence bases in a more considered fashion, including a more considered approach to guideline development and more conservative and rigorous implementation strategies. Coming back to SDD, large, high-quality, multi-national trials are currently underway testing the role of SDD in preventing deaths from sepsis whilst also studying the trade-off effects of SDD on antibiotic resistance [14, 15]. It does seem reasonable to hold any further implementation of SDD whilst these trials are completed.

## Conclusion

How we deal with the ultimate results of these SDD studies will act as the litmus test of whether we, as a specialty, have come of age.

## Abbreviations

RCT: Randomised controlled trials
SDD: Selective decontamination of the digestive tract
VAP: Ventilator associated pneumonias
